# Experimental Pharmacology in Transgenic Rodent Models of Alzheimer’s Disease

**DOI:** 10.3389/fphar.2019.00189

**Published:** 2019-03-04

**Authors:** A. Claudio Cuello, Hélène Hall, Sonia Do Carmo

**Affiliations:** ^1^Department of Pharmacology and Therapeutics, McGill University, Montreal, QC, Canada; ^2^Department of Neurology and Neurosurgery, McGill University, Montreal, QC, Canada; ^3^Department of Anatomy and Cell Biology, McGill University, Montreal, QC, Canada

**Keywords:** Alzheimer’s disease, cholinergic, DNA hypomethylation, experimental therapy, neuroinflammation, lithium, muscarinic/sigma1 receptors, S-adenosyl methionine

## Abstract

This Mini Review discusses the merits and shortfalls of transgenic (tg) rodents modeling aspects of the human Alzheimer’s disease (AD) pathology and their application to evaluate experimental therapeutics. It addresses some of the differences between mouse and rat tg models for these investigations. It relates, in a condensed fashion, the experience of our research laboratory with the application of anti-inflammatory compounds and S-adenosylmethionine (SAM) at the earliest stages of AD-like amyloid pathology in tg mice. The application of SAM was intended to revert the global brain DNA hypomethylation unleashed by the intraneuronal accumulation of amyloid-β-immunoreactive material, an intervention that restored levels of DNA methylation including of the *bace1* gene. This review also summarizes experimental pharmacology observations made in the McGill tg rat model of AD-like pathology by applying “nano-lithium” or a drug with allosteric M1 muscarinic and sigma 1 receptor agonistic properties (AF710B). Extremely low doses of lithium (up to 400 times lower than used in the clinic) had remarkable beneficial effects on lowering pathology and improving cognitive functions in tg rats. Likewise, AF710B treatment, even at advanced stages of the pathology, displayed remarkable beneficial effects. This drug, in experimental conditions, demonstrated possible “disease-modifying” properties as pathology was frankly diminished and cognition improved after a month of “wash-out” period. The Mini-Review ends with a discussion on the predictive value of similar experimental pharmacological interventions in current rodent tg models. It comments on the validity of some of these approaches for early interventions at preclinical stages of AD, interventions which may be envisioned once definitive diagnosis of AD before clinical presentation is made possible.

## Introduction

Alzheimer’s disease (AD) is a progressive neurodegenerative disease and the leading cause of dementia in the elderly (Hardy et al., 2014; Aisen et al., 2017). Postmortem brains from AD patients display characteristic pathological hallmarks, defined as extracellular amyloid plaques and intracellular neurofibrillary tangles made of hyper-phosphorylated tau protein. In addition, the cholinergic system is severely compromised. The vast majority of AD cases is sporadic, while <1% of cases corresponds to autosomal dominant forms, or familial AD (FAD), in which specific genetic mutations drive disease onset (Bateman et al., 2011; Ryman et al., 2014).

To date, there is no treatment to cure or prevent the development of the disease. Patients only have access to a few therapeutic options which offer limited symptomatic relief. Three of the four drugs approved by the Food and Drug Administration (FDA) for AD are cholinesterase inhibitors (AChEI). They enhance cholinergic synaptic transmission by preventing the breakdown of acetylcholine, a neurotransmitter essential for learning and memory. The fourth drug is memantine, an NMDA-receptor antagonist, while an additional pharmacological option combines one of the AChEI (donepezil) and memantine (Alzheimer’s Association, 2018).

Although studies would indicate a decrease in the incidence of AD in high-income countries over the past 10 years (Langa, 2015; Derby et al., 2017; Seblova et al., 2018), it is unlikely to offset the aging population. In fact, AD prevalence is increasing worldwide and is reaching near-epidemic proportions (Hickman et al., 2016). In that context, it is urgent to develop new drugs that can halt or prevent the progression of the disease. As of January 30, 2018, there were 112 agents in the AD treatment pipeline registered on clinicaltrial.gov, 63% of which are disease-modifying therapies (Cummings et al., 2018), illustrating the current efforts devoted to finding a cure for AD. Preclinical studies constitute an important cornerstone in drug development; validation of drug safety and efficacy in animal models is a necessary step before moving a drug into clinical trials. As such, animal models are a critical component to move the research field forward.

In this review, we will provide a concise overview of transgenic (tg) rodent models mimicking key aspects of the AD pathology and discuss their value in the drug development pipeline, as well as the challenges to predict drug efficacy in AD patients based on animal studies. In particular, we will focus on our experience with our transgenic mouse and rat models and describe experimental pharmacology studies using these models.

## Overview of Transgenic Models of Alzheimer’s Disease

A large number of tg mice and rats reproducing key features of the AD pathology have been generated. As of December 2018, the Alzheimer Forum Website lists 160 tg rodent models in its online database^1^ (Kinoshita and Clark, 2007), 156 of which are mice and only 4 are rats. The majority of these models express human genes bearing mutations described in FAD cases and human tauopathies, as well as human genes identified by Genome Wide Association Studies as susceptibility genes increasing the risk of developing AD (Tosto and Reitz, 2013). As such, they mostly mimic rare familial forms of AD and may not provide data translatable to sporadic AD. The most popular models are based on overexpression of human amyloid precursor protein (APP), presenilin (PSEN) [part of the γ-secretase complex involved in the cleavage of APP into amyloid-β (Aβ)] and tau, alone or in combination, to trigger accumulation of high levels of Aβ into plaques as well as the development of tauopathy (Spires and Hyman, 2005).

In particular, the most frequently used mouse models have been mice overexpressing human APP with the double Swedish (K670N and M671L) (Mullan et al., 1992) and the Indiana (V717F) (Murrell et al., 1991) mutations, such as the PDAPP (Games et al., 1995), tg2576 (Hsiao et al., 1996) and J20 (Mucke et al., 2000) models, as well as mice overexpressing mutant human APP and PSEN such as the APP/PS1 (Holcomb et al., 1998) and 5xFAD (Oakley et al., 2006) mice. One of the most popular mouse models to date is the 3xTg model harboring mutated APP, PSEN and tau genes (Oddo et al., 2003). In parallel, mouse models of tauopathies, in particular the hTauP301S (Allen et al., 2002) and PS19 mice (Yoshiyama et al., 2007), have become popular to study the tau component of the AD pathology (Gotz et al., 2007), although their main limitation resides in the expression of human tau harboring mutations seen in frontotemporal dementia and parkinsonism linked to chromosome 17 and not in AD. The particular characteristics and merits of tg mice for AD research have been discussed in several reviews (Hsiao, 1998; Bales, 2012; LaFerla and Green, 2012; Puzzo et al., 2015; Ameen-Ali et al., 2017; Jankowsky and Zheng, 2017; Sasaguri et al., 2017).

The most frequently used tg rats modeling the AD-like amyloid pathology are the McGill-R-Thy1-APP (Leon et al., 2010) and TgF344-AD (Cohen et al., 2013) rats. The clear imbalance in the ratio of mouse/rat models of AD reflects the inherent difficulty of producing tg rats in the early years of transgenesis, rather than an advantage of mice over rats. However, advances in transgenesis techniques have had a substantial impact on the development of tg rats (Filipiak and Saunders, 2006). In fact, rats are better suited than mice for biomedical research relevant to human. The increased value of rats over mice to study, in particular, human neurodegenerative conditions has been highlighted in recent reviews, with an emphasis on cognitive outcomes (Do Carmo and Cuello, 2013) and brain imaging (Zimmer et al., 2014). More recently, Drummond and Wisniewski (2017) discussed the relative characteristics of diverse species for a better representation of the human AD pathology and the challenges ahead.

Tg rodent models of AD have undoubtedly provided invaluable insights into the pathological processes of AD. They have also been instrumental in moving experimental therapy into clinical trials, following reports of positive therapeutic outcomes in rodent models. In this context, tg models played a pivotal role in the development of immunotherapy for AD, following the first experimental evidence that immunization of PDAPP mice with fibrillary Aβ_42_ decreased amyloid pathology (Schenk et al., 1999). Subsequent studies demonstrated that vaccination with Aβ also prevented memory loss in tg mouse models of AD (Janus et al., 2000; Morgan et al., 2000), which rapidly led to clinical trials. The first of these trials was unfortunately prematurely halted after a subset of patients developed acute meningoencephalitis (Orgogozo et al., 2003). Since then, more than one thousand publications have emerged illustrating efficacy, in varying degrees, of a large range of compounds. Some of these experimental attempts have been reviewed in LaFerla and Green (2012) and Sasaguri et al. (2017).

## Pharmacological Therapy in Mouse and Rat Models of the Ad-Like Amyloid Pathology – Insights From Our Experience

Our laboratory has developed tg lines expressing human APP with the double Swedish and the Indiana mutations under the Thy1 promoter, which recapitulate key features of the AD-like amyloid pathology in mice and rats. The McGill-Thy1-APP mice (Ferretti et al., 2011) and McGill-R-Thy1-APP rats (Leon et al., 2010) have been extensively studied by us (Cuello et al., 2010, 2012; Ferretti et al., 2012a,b; Hanzel et al., 2014; Iulita et al., 2014, 2017; Pimentel et al., 2015; Do Carmo et al., 2016, 2018; Wilson et al., 2017a,b, 2018; Hall et al., 2018) and others (Nilsen et al., 2012, 2014a,b; Galeano et al., 2014, 2018; Qi et al., 2014, 2018; Heggland et al., 2015; Martino Adami et al., 2017a,b; Parent et al., 2017; Zhang et al., 2017; Prestia et al., 2018). In the following section, we will discuss the particular value of these specific models in experimental pharmacology.

### McGill-Thy1-APP Mice

In the McGill-Thy1-APP mouse model, overexpression of mutant human APP triggers intraneuronal accumulation of Aβ material, including Aβ oligomers and fibrillary oligomers in cortical and hippocampal pyramidal neurons. Cognitive deficits are present prior to deposition of extracellular amyloid plaques at 4 months of age. At the same time, cholinergic alterations are detected in the cerebral cortex. Levels of insulin degrading enzyme, a well-established Aβ degrading enzyme, are downregulated in the cerebral cortex, suggesting that an impaired clearance of Aβ material likely contributes to the Aβ accumulation (Ferretti et al., 2011). In addition, microglial activation and recruitment toward Aβ-burdened neurons is present before plaque formation, along with an upregulation in major histocompatibility complex II, inducible nitric oxide (i-NOS) and CD40. This pre-plaque proinflammatory process is also accompanied by neuronal cyclooxygenase-2 (COX-2) upregulation (Ferretti et al., 2012b).

#### Minocycline

At late, clinical stages of AD, there is well-characterized chronic CNS inflammation. This late neuroinflammatory process is characterized by a strong immune response and phagocytic removal of Aβ material (McGeer and Rogers, 1992; McGeer and McGeer, 2013). These findings and the evidence that long term NSAIDs treatment for rheumathoid arthritis diminished AD prevalence (McGeer et al., 1990) sparked the idea that therapeutic agents directed at lowering inflammation could be of benefit for AD patients. However, studies showed that anti-inflammatories administered after AD clinical presentation did not improve cognitive decline (Jaturapatporn et al., 2012). In contrast, a large number of epidemiological studies confirmed that cognitively normal individuals receiving long term anti-inflammatory medication had a reduced risk of developing AD when compared to the general population (Breitner et al., 2011; McGeer and McGeer, 2013; Zhang et al., 2018). Taken together, these apparent contradictory findings support the idea that CNS inflammation at early (preclinical) and late (clinical) stages of AD are fundamentally distinct processes and suggest that blunting the early disease-aggravating inflammatory process may be of benefit for AD patients, concepts that have been further discussed in an opinion paper in TIPS (Cuello, 2017) and in a “white paper” in Alzheimer’s and Dementia by Rogers (2018).

As detailed in the previous section, McGill-Thy1-APP mice display an early pre-plaque disease-aggravating neuroinflammatory process (Ferretti et al., 2011) which, according to the above, may constitute an interesting target for therapeutic intervention. To test this hypothesis, and supported by prior observations (Seabrook et al., 2006), tg mice were treated with minocycline, a tetracyclic derivative with anti-inflammatory properties, at a young, pre-plaque stage, for one month. Minocycline treatment corrected the upregulation of i-NOS and COX-2, lowered interleukin-1β levels and decreased microglial activation. The reduction in inflammation was accompanied by changes in the APP processing, namely a decrease in full length APP and Aβ trimer levels, while the activity of the β-site APP cleaving enzyme 1 (BACE1) was normalized (Ferretti et al., 2012a). Overall this study demonstrates that the early, pre-plaque, disease aggravating neuroinflammatory process triggered by the AD-like amyloid pathology can be dampened by the pharmacological application of minocycline.

These findings would support the notion that targeting inflammation early in the disease process should have a beneficial outcome in individuals with preclinical AD pathology. Successful implementation of such a strategy will, however, rely on the unequivocal identification of biomarkers of AD preclinical stages.

#### S-Adenosylmethionine (SAM)

There is growing evidence revealing that epigenetic deregulations and dysregulated DNA methylation may unleash disease-aggravating mechanisms in several neuropathologies. AD patients and AD animal models display global and gene-specific DNA hypomethylation within the brain (West et al., 1995; Mastroeni et al., 2010; Bakulski et al., 2012; Chouliaras et al., 2013; Cadena-del-Castillo et al., 2014; De Jager et al., 2014; Iwata et al., 2014; Smith et al., 2016; Nicolia et al., 2017; Zhao et al., 2017), likely contributing to the AD pathology and associated memory deficits. We have demonstrated the occurrence of global DNA hypomethylation, which is prominent in neurons, at early pathological stages in McGill-Thy1-APP mice. Importantly, the *bace1* promoter is hypomethylated in our tg mice. This was associated with higher levels of BACE1 protein and BACE1 activity, and in consequence increased Aβ peptides in the CNS (Do Carmo et al., 2016).

The cause of global hypomethylation in AD remains unclear. However, we found that early intraneuronal accumulation of Aβ is sufficient to provoke global DNA hypomethylation (Do Carmo et al., 2016). Importantly, in AD patients the levels of the ubiquitous methyl donor S-adenosylmethionine (SAM) are low in the brain and cerebrospinal fluid (Bottiglieri et al., 1991; Morrison et al., 1996). This prompted us to administer systemically SAM to McGill-Thy1-APP mice, starting at early stages of the pathology. This straightforward therapy was sufficient to abolish the pre-existing global hypomethylation, as well as *bace1* hypomethylation. BACE1 protein levels and activity were restored to control levels and brain amyloid pathology decreased. This strategy was sufficient to fully revert the cognitive impairment consequent to the CNS amyloid pathology, as revealed by the novel object recognition (NOR) and the Morris water maze (MWM) tests (Do Carmo et al., 2016). The findings, in our model, of an early neuronal demethylation and its rescue by SAM treatment reinforce a causal link between Aβ accumulation and impaired DNA methylation, rather than the consequence of stochastic events.

In addition, in human brains from the Religious Orders Study cohorts, we also found correlations between *bace1* methylation levels and amyloid and tangle load as well as measures of cognition (Do Carmo et al., 2016). Overall, our observations in tg mice and AD patients suggest that DNA hypomethylation should be considered as a drugable target for the treatment of AD, and that, in consequence, SAM-based therapy might constitute a promising therapeutic avenue. Of note, the use of SAM in the treatment of multiple neuropsychiatric disorders is currently being explored (Sharma et al., 2017).

### McGill-R-Thy1-APP Rats

The McGill-R-Thy1-APP rats display early intraneuronal accumulation of Aβ in the hippocampus and cerebral cortex, evident one week after birth. As the amyloid pathology progresses with age, extracellular plaques start to develop. The first plaques are observed in the subiculum at 6–9 months of age (Leon et al., 2010) and later spread through anatomically connected regions (Heggland et al., 2015). Cognitive deficits are apparent at 3 months of age and worsen as the pathology progresses (Leon et al., 2010). This evolving pathology is accompanied by an early, disease-aggravating, pre-plaque neuroinflammatory process (Hanzel et al., 2014). Deficits in synaptic plasticity are evident at the pre-plaque stage and appear to be inflammasome-dependent (Qi et al., 2014, 2018). Similarly, deficits in synaptosomal bioenergetics are reported before the appearance of plaques (Martino Adami et al., 2017b). At the post-plaque stage, the NGF metabolic pathway is dysregulated and shows impairment in neurotrophin expression (Iulita et al., 2017), as seen during the progression of AD in patients (Iulita and Cuello, 2016). These changes are accompanied by a reduction in cholinergic synaptic boutons (Iulita et al., 2017). Moderate neuronal loss in the subiculum (Heggland et al., 2015), hippocampal shrinkage and glucose hypometabolism further characterize the post-plaque stage (Parent et al., 2017). Overall, in the McGill-R-Thy1-APP rat model, a slowly evolving amyloid pathology triggers a cascade of events reminiscent of what is seen in AD brains, thereby offering an array of targets possibly amenable to therapeutic intervention.

#### NP03-Lithium

The complex nature of the human AD pathology, which involves multiple processes such as abnormal Aβ and tau processing, CNS inflammation, mitochondrial dysfunction and calcium dyshomeostasis, calls for the use of multi-target drugs rather than a compound targeting a single molecule. In that context, lithium is of interest as it reportedly has the ability to modulate several of these pathways (Malhi and Outhred, 2016).

Lithium salts are widely used in the treatment of psychiatric conditions such as bipolar disorder. A limited number of studies have also examined the potential use of lithium in amnestic mild cognitive impairment and clinical AD populations, and have reported promising but often conflicting results (Havens and Cole, 1982; Terao et al., 2006; Macdonald et al., 2008; Hampel et al., 2009; Leyhe et al., 2009; Forlenza et al., 2011, 2016; Nunes et al., 2013; Mauer et al., 2014; Morris and Berk, 2016; Kessing et al., 2017). Unfortunately, lithium has a narrow therapeutic window and a low brain penetration. It elicits with some frequency severe side effects which limit its long-term use in the elderly population (Gelenberg and Jefferson, 1995; Livingstone and Rampes, 2006; Azab et al., 2015). Coincidently with the finding that trace amounts of lithium in drinking water are associated with a reduced incidence of dementia (Mauer et al., 2014; Kessing et al., 2017; McGrath and Berk, 2017; Fajardo et al., 2018), there has been a growing interest for low-dose lithium in the treatment of AD (Nunes et al., 2013, 2015; Zhao et al., 2014). Treatment with microdose lithium was shown to prevent cognitive decline in AD patients and in AD tg models (Nunes et al., 2013, 2015).

NP03 is a novel formulation of lithium, in which microdose lithium is encapsulated in a water-in-oil microemulsion (Aonys technology developed by Medesis Pharma, Montpellier, France). This formulation can enhance the CNS penetration of significantly lower amounts of lithium, with doses 100 to 400 times lower than what is usually prescribed for bipolar disorder. Such doses can most likely avoid the adverse effects associated with classical, higher dosage, lithium formulations. Treatment of young, pre-plaque McGill-R-Thy1-APP rats with NP03 resulted in cognitive improvements as measured by the NOR, MWM and fear conditioning tests. Unexpectedly, NP03 treatment also reduced BACE1 levels and activity, leading to a decrease in levels of soluble Aβ42. NP03 treatment was also shown to inactivate GSK-3β, rescue impaired CRTC1 promoter binding of synaptic plasticity genes and restore hippocampal neurogenesis in tg rats (Wilson et al., 2017b). In addition, NP03 reduced oxidative stress and inflammation markers in tg rats, as evidenced by decreased levels of protein-bound 4-hydroxynonenal and protein-resident 3-nitrotyrosine, as well as reduced production of cytokines such as TNF-α, IFN-γ, IL-10, and IL-5. Finally, NP03 downregulated transcripts levels of the microglia surface receptor TREM2 concomitantly with a reduced recruitment of microglia toward Aβ-burden neurons in the hippocampus (Wilson et al., 2018). These findings highlight the value of novel formulations of non-toxic microdose lithium NP03 in the treatment of AD at its early, preclinical, stages.

#### Combined M1 Muscarinic/Sigma 1 Receptor Agonist

In the early 1990s, a pivotal study demonstrated that activation of muscarinic cholinergic receptors could modulate the APP processing toward a non-amyloidogenic pathway (Nitsch et al., 1992), thereby reducing the production of toxic Aβ. It was later shown that the M1 muscarinic receptors in particular played a key role in modulating the AD-like amyloid pathology *in vivo* (Caccamo et al., 2006). Following these seminal discoveries, it was hypothesized that M1 receptor agonists could extend the cholinergic-based therapeutic arsenal for AD: they should, not only enhance the cholinergic tone for the cognitive benefits of AD patients, but also provide disease-modifying properties. Unfortunately, first generation muscarinic agonists lacked specificity for the M1 receptors, and activation of other receptor subtypes led to major side effects (Fisher, 2008). However, the generation of novel subtype specific muscarinic agonists has renewed the interest for this type of compounds (Fisher, 2012).

Given the complexity of AD pathophysiology, drugs that can target multiple receptors or impaired signaling pathways would likely offer a therapeutic advantage over more conventional single-target drug, as illustrated in the previous section. In that regards, the novel compound AF710B (aka ANAVEX 3-71) merits further attention. It is a combined selective allosteric M1 muscarinic and sigma 1 receptor agonist (Fisher et al., 2016). Targeting sigma 1 receptor has been shown to provide neuroprotection and anti-amnestic properties (Marrazzo et al., 2005; Maurice and Su, 2009; Villard et al., 2011). Combined with M1 muscarinic receptor-mediated effects on APP metabolism, it would confer AF710B a pharmacological profile of interest to tackle various pathological aspects of AD.

AF710B can rescue synapse loss *in vitro*, while low doses of the compound can attenuate cognitive deficits and alleviate hallmarks of the established AD-like pathology in 3xTg-AD mice (Fisher et al., 2016). In order to validate the putative disease-modifying effect of the drug, AF710B was administered (in the micromolar range) *per os* daily to post-plaque McGill-R-Thy1-APP rats for 5 months. Completion of the treatment was followed by a wash-out phase of 5 weeks, a unique experimental design key to discriminate true disease-modifying effects from symptomatic effects. The former should disappear after treatment cessation while the latter would be persistent (Ploeger and Holford, 2009). This treatment regimen was sufficient to fully restore cognition in the McGill tg rats. It also led to a substantial decrease in the production of cortical Aβ and in the amount of mature amyloid plaques. Interestingly, the reduction in Aβ load was accompanied by an increase in CSF Aβ42 levels, suggesting that the drug could not only lower Aβ production but also increase its clearance (Hall et al., 2018). Of note, this finding may be of translational value to non-invasively follow treatment response in a human population. Further to it, CNS inflammation was decreased, as mirrored by the abolishment of microglia recruitment toward Aβ-burdened neurons in the hippocampus and the normalization of hippocampal Iba1 protein levels and cortical IL-10 mRNA expression levels. Finally, these changes were accompanied by an increase in synaptophysin levels, suggesting possible synaptogenic activity (Hall et al., 2018).

In summary, with M1/sigma-1 activity as well as putative disease-modifying properties at very low dose, AF710B is well positioned for therapeutic interventions in AD. The case of AF710B as detailed above provides interesting clues pertaining to potentially increasing the predictive value of preclinical studies. In this particular case, results from the study in McGill-R-Thy1-APP tg rats complemented the initial findings of AF710B beneficial effects in trihexyphenidyl rats and tg mice, by offering new insights into the properties of the drug (Fisher et al., 2016; Hall et al., 2018). Such approach, where efficacy of a drug is validated in several models and under different conditions, should be encouraged before translation to human trials. This is particularly important considering that no single model can recapitulate all aspects of the human disease.

## General Considerations: Translational Value of Experimental Pharmacology in Rodent Models

Despite the growing list of compounds that have demonstrated positive therapeutic effects in tg rodent models, none of these experimental leads have yet reached FDA-approval for the treatment of AD in humans, highlighting a clear deficit in translational research. In line with these failures, the question that arises is: how can we better predict drug efficacy in humans based on animal studies?

Although tg APP rodent models have been instrumental in increasing our understanding of Aβ-driven pathogenic processes in AD, it is challenging to ascertain whether the phenotypes observed in these tg animals can be solely attributed to elevated Aβ levels or result from overexpression of APP. To address this concern, single humanized APP knock-in (KI) mice have been recently developed (Saito et al., 2014), paving the way toward more physiological models of the disease. Evidently, one of the main shortcomings of the current tg AD models is that they express genes carrying mutations seen in FAD, whereas most AD cases are sporadic. It remains an open question whether the findings obtained from FAD-like models can in fact be translated to heterogeneous sporadic cases, in which a multitude of susceptibility factors likely drive disease onset (genetic, environmental or lifestyle) (Rocchi et al., 2003; Mattson, 2004).

Mouse has been the species of choice since the inception of tg models, mostly for technical and economic reasons. Several characteristics unique to rats would, however, indicate that they may be better suited than mice for research relevant to humans, especially in the neuroscience field [reviewed in Do Carmo and Cuello (2013); Zimmer et al. (2014) and Ellenbroek and Youn (2016)]. Indeed, rats have a more complex CNS than mice and their brain development in postnatal life is more similar to humans than mice (Wood et al., 2003; Pressler and Auvin, 2013). Although the rat and mouse brains are anatomically similar, there are important functional differences between the two, including substantial differences in neuronal plasticity. For example, recent studies indicate that in adult rats, the rate of hippocampal neurogenesis is faster than in mice and new neurons are more likely to be recruited during learning than in mice (Snyder et al., 2009). Rats display a richer behavior including more complex social behavior (such a juvenile play) compared to mice, allowing for a larger range of cognitive analyses (Whishaw et al., 2001; Do Carmo and Cuello, 2013). Genetically, they also resemble humans more than mice do. For example, the rat apolipoprotein E (ApoE) gene bears more homology with human ApoE than mouse ApoE (Rajavashisth et al., 1985; Tran et al., 2013). This is of significance considering that polymorphism in ApoE is a strong genetic risk factor for AD, with ApoE4 showing the strongest association with the disease (Poirier et al., 1993; Strittmatter et al., 1993; Roses et al., 1994). As the genetic toolbox for rats is growing, the number of tg rat models will likely increase in the future. Whether rat-based models will better predict the human condition and the efficacy of experimental therapeutics remains to be established.

One important aspect of current tg models is that they likely mimic early disease stage when the focus of most current clinical trials has been mild-moderate AD. It is therefore highly likely that some of the therapeutic leads emerging from experimentation in tg mice and rats might have an important impact in slowing or diminishing the AD pathology at the preclinical stage. Such opportunity would have a consequential impact on the global AD prevalence since it has been estimated that a 5-year delay in the onset of clinical AD would decrease the total number of affected individuals by 50% at the end of a 25 year period (Alzheimer’s Association, 2015). However, adequate identification and selection of human participants for such trials remains challenging and is pending on the discovery of novel biomarkers that can unequivocally identify preclinical AD stage. In the meantime, some clinical trials have been focusing on individuals with a high risk of developing AD dementia due to a family history of AD. These individuals have been recruited and enrolled before they develop any sign of cognitive decline (Cummings et al., 2018). Results from these ongoing prevention trials such as the DIAN-TU, DEPEND or HEART studies (which have enrolled individuals with a family history of autosomal dominant AD, a family history of AD or a parental history of AD, respectively) are highly anticipated and may indicate whether pharmacological interventions at preclinical AD stages could prevent or delay cognitive decline.

## Conclusion

Over the past decades, our understanding of AD has grown tremendously, owing for a large part to the contribution of tg models mimicking various aspects of the AD pathology. However, despite intensive research, there is still no cure for this devastating disease. As the prevalence of AD increases worldwide, it is crucial to identify bottlenecks in the drug development pipeline, which may slow down progress toward the market approval of promising drug candidates. The shortcomings of current tg models of AD are well-acknowledged by the research community. In response, there is a growing effort to provide better predictive animal models of the disease. Along these lines, current efforts toward the identification of biomarkers that would identify an ongoing AD process before clinical presentation will most likely culminate in refined clinical trial design. These initiatives are much needed to translate positive preclinical studies into efficacy in human clinical trials. An optimist outlook regarding successful preclinical studies is that they might offer novel therapeutic avenues with probable tangible benefits if applied at the earliest, preclinical stages of the disease. A favorable future scenario in which diagnosis of AD pathology is made about 10 years before dementia could help radically change the currently disappointing therapeutic arsenal.

## Author Contributions

ACC and HH designed the structure and contents of the review. All authors contributed to the writing of the manuscript.

## Conflict of Interest Statement

The authors declare that the research was conducted in the absence of any commercial or financial relationships that could be construed as a potential conflict of interest.

## Footnotes

1 https://www.alzforum.org/research-models/alzheimers-disease
